# Impact of the systematic introduction of low-cost bubble nasal CPAP in a NICU of a developing country: a prospective pre- and post-intervention study

**DOI:** 10.1186/s12887-015-0338-3

**Published:** 2015-03-25

**Authors:** Rossano Rezzonico, Letizia M Caccamo, Valeria Manfredini, Massimo Cartabia, Nieves Sanchez, Zoraida Paredes, Patrizia Froesch, Franco Cavalli, Maurizio Bonati

**Affiliations:** NICU Rho Az. Ospedaliera “G. Salvini” Garbagnate Milanese, Milan, Italy; Az. Ospedaliera Universitaria L. Meyer, Florence, Italy; UCIN Hospital Bertha Calderon Managua, Managua, Nicaragua; Department of Public Health, Laboratory for Mother and Child Health, IRCCS – Istituto di Ricerche Farmacologiche Mario Negri, Milan, Italy; AMCA, Aiuto Medico Centro America, Giubiasco, Switzerland

## Abstract

**Background:**

The use of Nasal Continuous Positive Airway Pressure Ventilation (NCPAP) has begun to increase and is progressively replacing conventional mechanical ventilation (MV), becoming the cornerstone treatment for newborn respiratory distress syndrome (RDS). Howerver, NCPAP use in Lower-Middle Income Countries (LMICs) is poor. Moreover, bubble NCPAP (bNCPAP), for efficacy, cost effectiveness, and ease of use, should be the primary assistance technique employed in newborns with RDS.

Objective: To measure the impact on in-hospital newborn mortality of using a bNCPAP device as the first intervention on newborns requiring ventilatory assistance.

**Methods:**

Design: Prospective pre-intervention and post-intervention study.

Setting: The largest Neonatal Intensive Care Unit (NICU) in Nicaragua.

Participants: In all, 230 (2006) and 383 (2008) patients were included.

Intervention: In May 2006, a strategy was introduced to promote the systematic use of bNCPAP to avoid intubation and MV in newborns requiring ventilatory assistance. Data regarding gestation, delivery, postnatal course, mortality, length of hospitalisation, and duration of ventilatory assistance were collected for infants assisted between May and December 2006, before the project began, and between May and December 2008, two years afterwards.

Outcome measures: The pre- vs post-intervention proportion of newborns who died in-hospital was the primary end point. Secondary endpoints included rate of intubation and duration of NICU stay.

**Results:**

Significant differences were found in the rate of intubation (72 vs 39%; p < 0.0001) and the proportion of patients treated exclusively with bNCPAP (27% vs 61%; p <0.0001). Mortality rate was significantly reduced (40 vs 23%; p < 0.0001); however, an increase in the mean duration of NICU stay was observed (14.6 days in 2006 and 17.5 days in 2008, p = 0.0481).

The findings contribute to the evidence that NCPAP, particularly bNCPAP, is the first-line standard of care for efficacy, cost effectiveness, and ease of use in newborns with respiratory distress in LMICs.

**Conclusions:**

This is the first extensive survey performed in a large NICU from a LMICs, proving the efficacy of the systematic use of a bNCPAP device in reducing newborn mortality. These findings are an incentive for considering bNCPAP as an elective strategy to treat newborns with respiratory insufficiency in LMICs.

## Article summary

### Article focus

- In Lower-Middle Income Countries (LMICs), a majority of the under-5 mortality rate is due to neonatal deaths. Evidence presents the Nasal Continuous Positive Airway Pressure ventilation (NCPAP) as an effective, low-cost, and easy-to-use intervention for enhancing newborn survival.
- The objective of this study was to evaluate the effects of using a bubble NCPAP (bNCPAP) device as the first intervention on mortality of newborns in the largest Neonatal Intensive Care Unit (NICU) in Nicaragua.

### Key messages

- The early use of bNCPAP as the primary respiratory assistance strategy was associated with a halved mortality rate in newborns in NICUs, and positive changes in medical and nursing assistance were notable.

### Strengths and limitations of this study

- This is the first wide-scale study demonstrating the efficacy of the systematic use of a low-cost bNCPAP device in a large NICU from one of the less developed countries. The study was conducted in a natural setting where clinical research and audit studies are not common, and was therefore also a practical, educational initiative.
- The main limitations were that no risk index to state the level of clinical severity of newborns could be applied and that there was a lack of both data regarding maternal socio-economic characteristics and of a thorough analysis of the medical costs.

## Background

A worldwide decrease in child mortality has been achieved in the last few years, following the global attempt to pursue Millennium Development Goal 4 (MDG4) adopted at the UN Millennium Summit in 2000, targeting a 2/3 reduction in under-5 mortality by 2015. In the last decade, however, the neonatal death rate seems to have increased from the 37% observed in 2000 to the 42% of the overall estimated child mortality recorded in 2010 [1]. Preterm birth and related complications are important leading causes of neonatal mortality.

In Lower-Middle Income Countries (LMICs), newborn deaths are dramatically prevalent, and lack of human and economical resources makes accomplishing the MDG4 challenging [2]. Among the interventions for reducing the newborn mortality rate, Nasal Continuous Positive Airway Pressure Ventilation (NCPAP) is of proven efficacy [3]. The bubble NCPAP (bNCPAP), is the simplest, low-cost respiratory support system, showing theoretical and experimental advantages, as well as similar, or greater, clinical efficacy compared to other, more sophisticated methods to deliver NCPAP (variable flow and ventilator NCPAP) [4-9].

In addition to positive experiences from Scandinavian countries [10], data published by the Neonatology Committee for the Developmental Epidemiology Network in 2000 have shown a significant inverse relationship between incidence of Bronchopulmonary Dysplasia (BPD) and use of NCPAP in very low birth weight (VLBW) newborns [11]. NCPAP therefore seems to offer an improvement compared to mechanical ventilation (MV) in this population. This was the starting point for various, important trials: Surfactant Positive Pressure and Pulse Oximetry Randomized Trial (SUPPORT) [12], Cpap Or Intubation (COIN) [13], Vermont Oxford Network (VON) [14], sURfactant and early nasal continuous Positive Airway Pressure (CURPAP) [15], Colombian Network [16], and Neocosur Network on early use of NCPAP [17]. As reviewed by W. Carlo, this “gentle ventilation”, when started soon after birth, appears to reduce the need for MV and the risk of BPD/death, and is an alternative to the prophylactic or early surfactant approach in low, very low, and extremely low birth weight infants (ELBW) [8-10,18-22].

The use of NCPAP has begun to increase and is progressively replacing conventional MV, becoming the cornerstone treatment for newborn respiratory distress syndrome (RDS).

The statistical power of the few very interesting studies addressing NCPAP use in LMICs is poor due to the small sample sizes. All studies agree that NCPAP, particularly bNCPAP, for efficacy, cost effectiveness, and ease of use, should be the primary assistance technique employed in newborns with RDS [3,8,9,23-27].

Nicaragua is the second poorest country in Central America and in 2009 the under-5 child mortality was 27/1000 newborns (8/1000 in the USA), with a maternal mortality of 100/100,000 live births. Among all causes of under-5 mortality, in 2008, 45% was due to neonatal causes, of which 22% were related to prematurity, 13% to congenital anomalies, 8% to asphyxia, and 2% to sepsis [28,29].

The Bertha Calderon Hospital (BCH), in Managua, is the biggest maternity hospital in Nicaragua, accounting for more than 10.000 deliveries and 600 admissions in the neonatal intensive care unit (NICU) per year.

AMCA (Aiuto Medico Centro America) is a Swiss non-governmental organisation that has been operating since 1985 [30] in Central America, particularly in Nicaragua and El Salvador. From 2005 to 2007 it set up, with the local NICU staff, a programme covering newborn resuscitation training courses, based on international guidelines [31], pain and O_2_ therapy-related risks control, and reduction in number of newborns on MV through the use of the bNCPAP device. A prospective study was thus performed in the largest NICU in Nicaragua to determine the impact of the early use of low cost bNCPAP to reduce intubation and MV and to determine its effects on mortality rate and duration of NICU stay.

## Methods

This study included newborns with a history of ventilatory assistance (VA) admitted to the BCH NICU during the periods 1 May 2006 to 31 December 2006 and 1 May 2008 to 31 December 2008. During 2007, AMCA experts provided BCH staff with an extensive course on bNCPAP use, which was repeated thereafter by local, specifically trained staff. The impact of the intervention was therefore measured comparing the period before the project began (2006) with that two years after the project’s start (2008).

The exclusion criteria were as follows: birth malformations, transfer to another hospital, suboptimal patient assistance due to lack of devices, and parental opposition to ventilation.

Data were collected from the infants’ medical records by a doctor not involved in the study. Data included delivery mode (vaginal = VAG *or* caesarean section = CS), gender (M/F), gestational age (GA) and birth weight (BW), main diagnosis at recovery, mean duration of NICU stay, recovery outcome, prenatal steroids, Apgar index, resuscitation measures, surfactant use, and age at start, and duration, of respiratory assistance (hours).

**Figure 1:**
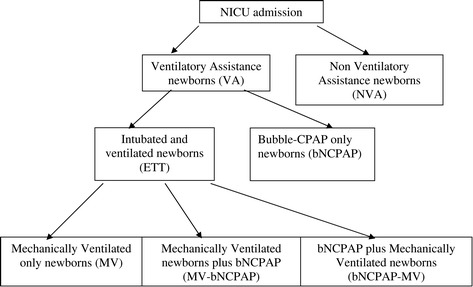
**Flowchart showing patient distribution.**

The study population was divided into groups: 1) those receiving only intubation and MV (MV group); 2) only bNCPAP (bNCPAP) 3) bNCPAP first, then MV (bNCPAP-MV); and 4) MV first, than bNCPAP (MV-bNCPAP). The groups of patients requiring MV alone or with bNCPAP (MV, bNCPAP-MV, MV-bNCPAP) were categorised into an additional group called endotracheal tube (ETT) (Figure 1).

The institutional boards of both the BCH and the IRCCS – Istituto di Ricerche Farmacologiche Mario Negri, Milan, Italy approved the study. Informed consent was obtained from the parents of the infants undergoing ventilation, in accordance with established practice.

### Resuscitation and ventilatory assistance

During the study, delivery room local medical staff provided resuscitation and ventilatory assistance. Newborn resuscitation was performed according to the American Academy of Pediatrics and American Heart Association guidelines [31].

No protocols or severity scores (eg. CRIB II) were defined during the study period due to lack of medical devices such as pulse oxymeters, blood gases analysers, and radiographic equipment in the delivery room [32]. The decision to start or to continue ventilatory assistance with MV or bNCPAP was based on clinical and anamnestic criteria, according to practices aimed at limiting MV, and “the need for supplemental oxygen in newborn infants [26].

No substantial changes in delivery room assistance or medical or nursing staff were made during the study period.

bNCPAP is a highly sophisticated, fluid-dynamic system in which the bubbles produced by the expiratory water valve improve lung mechanical characteristics and minimise the respiratory system impedance [7,12,33].

bNCPAP kits were provided to the NICU and included a humidifier and re-sterilisable breathing circuits (Fisher & Paykel, New Zealand) and, to minimise infection risks, disposable nasal cannula (Hudson, USA). To further reduce costs, a dual Air/Oxygen flowmeter (Harol, Italy) was chosen as the blender, because it is much less expensive than automatic blenders and provides results of similar quality.

### Statistical analysis

Microsoft Office Excel 2007 and SAS system software, release 8.2, were used for analysis. Values are presented as counts and percentages, mean ± standard deviation, or Odd’s ratios (OR) with 95%-confidence intervals (95% CI).

A bivariate analysis was performed to evaluate the differences observed between the two groups of ventilated patients (2006 *vs* 2008). For binary variables the chi-square test was used, while for continuous variables the t-test was used.

GA and BW variables were grouped into classes and transformed from quantitative into qualitative variables. For all the statistical tests a p-value <0.05 was deemed significant.

A multivariate logistic regression model, with the stepwise selection option, was performed including all the following covariates assessing the death-risk determinants in VA patients: period of admission (2006 or 2008), type of ventilatory assistance (bNCPAP or ETT), sex, GA (weeks) z-score, BW (g) z-score (z-scores were used since birth weight was highly collinear with gestational age), mode of delivery, prenatal steroid, 1-minute Apgar score, 5-minute Apgar score, resuscitation, and intubation.

Lastly, the mean duration of NICU stay and of hours of MV and bNCPAP were calculated and compared with t-tests in the two periods of interest.

## Results

A total of 651 newborns made up the study population from the periods analysed (248 in 2006 and 403 in 2008). Of these, 18 (7.3%) in 2006 and 20 (5.0%) in 2008 (p = 0.2251) did not fit inclusion criteria, leaving 613 VA newborns in the study (230 in 2006 and 383 in 2008).

### VA newborn characteristics

The characteristics of the patients from the two groups, described in Table 1, were all comparable except for a greater proportion of CS delivery patients during 2008 (53% in 2006 and 69% in 2008, p = 0.0001). No patients received surfactant treatment. In 2008 a lower proportion of VA patients received resuscitation (54% in 2006 *vs* 39% in 2008, p = 0.0006). The 1st and 5th minute Apgar indices were comparable in newborns who received resuscitation at birth (RES). A higher proportion of patients required intubation at birth in 2006 (p = 0.0410). No differences were observed in rate of steroid prophylaxis use.

**Table 1:** **Characteristics of the newborns with ventilatory assistance (VA)**

	**2006**		**2008**		**p**
VA patients N (%)	230	(74.7)	383	(81.3)	0.0269
Sex M/F ratio	142/88	1.61	238/145	1.64	ns
CS/VAG ratio	121/109	1.11	261/119	2.19	<0.0001
Prenatal steroids N (%)	73	(33.6)	119	(31.6)	ns
GA weeks, N mean ± [SD]	216	34.3 ± [3.9]	379	34.4 ± [3.8]	ns
<27 N (%)	8	(3.7)	18	(4.7)	ns
28-33 N (%)	73	(33.8)	112	(29.6)	ns
34-36 N (%)	70	(32.4)	135	(35.6)	ns
37-42 N (%)	63	(29.2)	114	(30.1)	ns
>42 N (%)	2	(0.9)	-	-	ns
BW g, N mean ± [SD]	217	1975 ± [792]	377	2001 ± [766]	ns
ELBW N (%)	14	(6.5)	38	(10.1)	ns
VLBW N (%)	58	(26.7)	73	(19.4)	0.0371
LBW N (%)	87	(40.1)	161	(42.7)	ns
NBW N (%)	58	(26.7)	105	(27.9)	ns
RES /VA	116/216	(53.7)	147/376	(39.1)	0.0006
1-minute Apgar RES N, mean + [SD]	116	5.1 ± [2.4]	144	5.5 ± [2.2]	ns
5-minute Apgar RES N, mean + [SD]	116	6.9 ± [2.5]	144	7.2 ± [2.1]	ns
Intubated at birth / RES (%)	29/116	(25.0)	22/147	(15.0)	0.0410
Death N (%)	93	(40.4)	87	(22.7)	<0.0001

CS Caesarean section delivery.

VAG Vaginal delivery.

RES Newborns resuscitated at birth.

NICU admission diagnoses, despite the aforementioned limitations, were comparable in 2006 and 2008 (Table 2).

**Table 2:** **Comparison of recovery diagnoses**

**Diagnoses**	**2006 (N)**	**2008(N)**	**2006 (%)**	**2008 (%)**
Unknown	6	10	2.6	2.6
Apnoea	8	14	3.5	3.7
Asphyxia	13	19	5.7	5.0
Seizure	2	3	0.9	0.8
RDS	185	311	80.4	81.2
Sepsis and shock	15	26	6.5	6.8
Polycythaemia	1	0	0.4	0
Total	230	383	100	100

RDS includes: pulmonary bleeding, congenital pneumonia, prematurity RDS, MAS, transient neonatal tachypnoea, pulmonary emphysema.

### Ventilatory assistance mode

In 2006 the MV patient prevalence was 50% and decreased to 10% in 2008 (p <0.0001). During the two periods, no differences were found in the proportion of MV-bNCPAP patients (p = 0.5467), while a significant increase from 15% to 23% (p = 0.0202) was found in the bNCPAP-MV group. The rate of ETT patients was 72% in 2006 and decreased to 39% in 2008 (p <0.0001).

**Figure 2:**
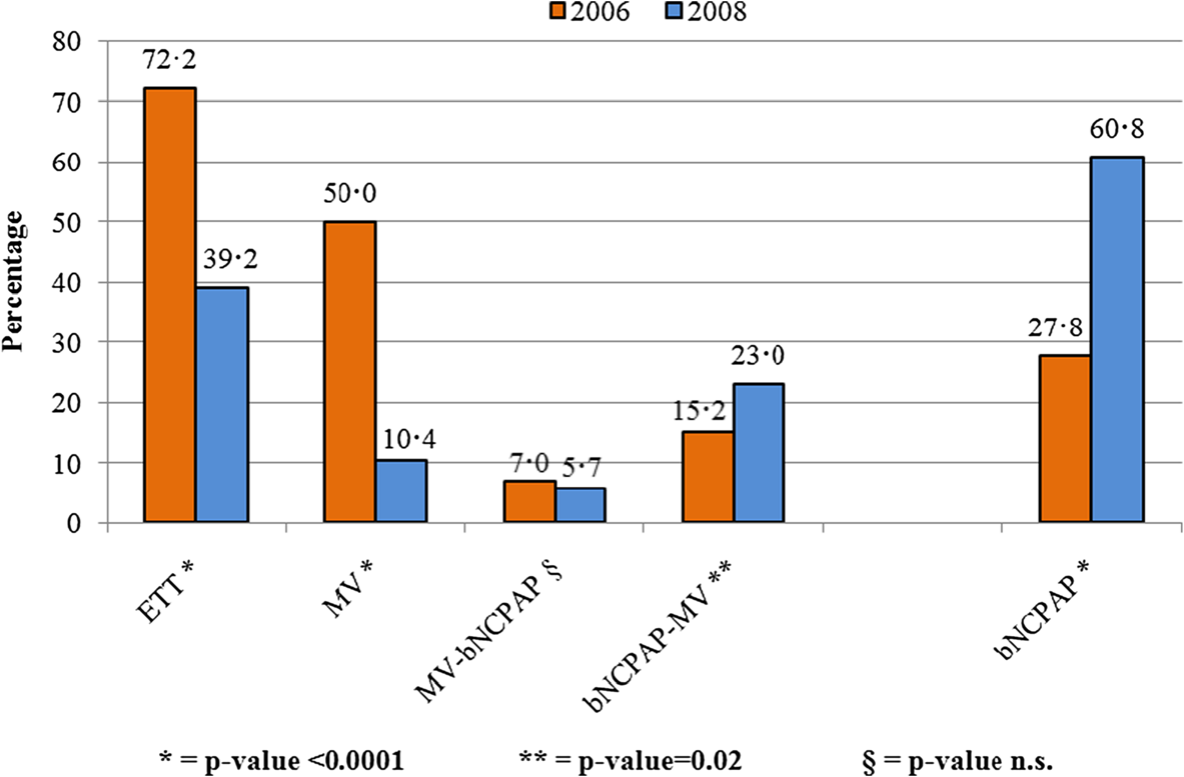
**Ventilatory assistance newborns and ventilation type.**

The introduction of bNCPAP led to an increase newborns treated with this device, from 28% in 2006 to 61% in 2008 (p <0.0001), consistent with the decrease in ETT patients (Figure 2).

In 2008, 54% of the bNCPAP newborns started treatment at birth, 74% within the first, and 86% within the second hour of life.

### Mortality

**Figure 3:**
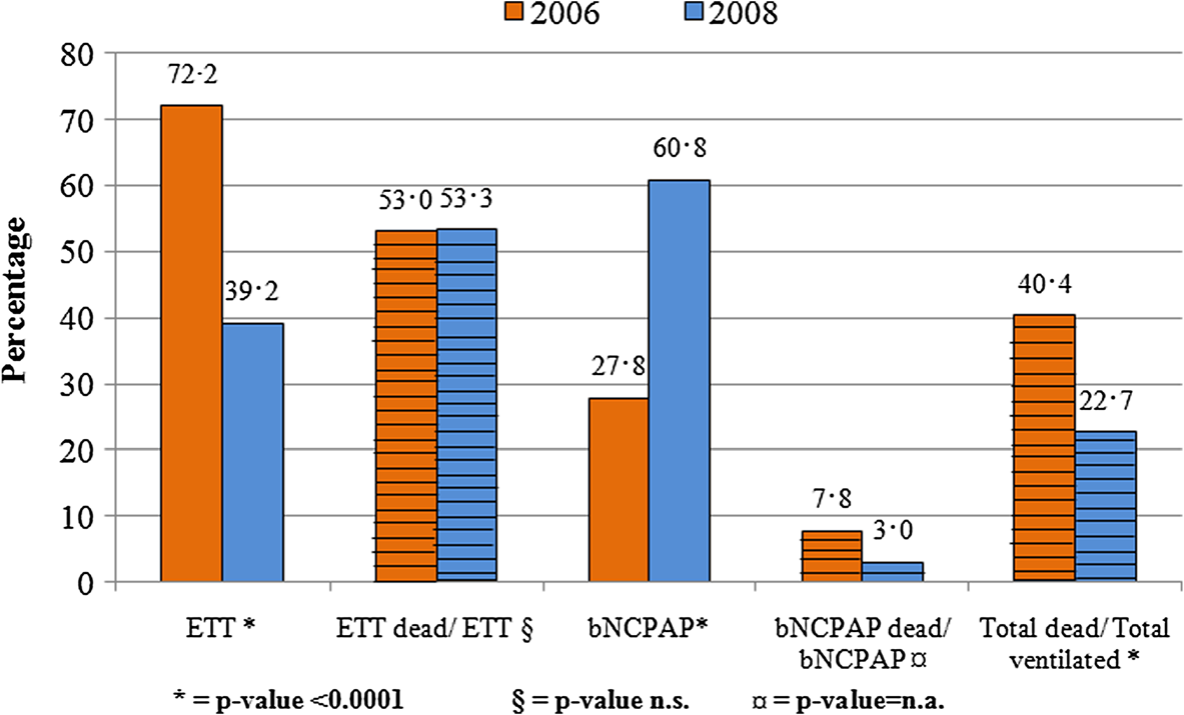
**Distribution by ventilation type and related deaths (striped).**

In 2008 the number of VA patients admitted to the NICU increased by 67%, but mortality decreased significantly, from 40.4% (n = 93) in 2006 to 22.7% (n = 87) (p < 0.0001). In ETT newborns, the mortality rate remained unchanged, from 53.0% to 53.3% (p = ns) (Figure 3). If only the MV patient group was considered, however, mortality increased from 52% in 2006 to 90% in 2008 (p < 0.0001) (Table 3).

**Table 3:** **Mortality rate distribution**

	**2006**		**2008**		**p**
Deaths VA	93/230	(40.4)	87/383	(22.7)	<0.0001
Deaths ETT	88/166	(53.0)	80/150	(53.3)	ns
Deaths MV	60/115	(52.2)	36/40	(90.0)	<0.0001
Deaths MV-NCPAP	7/16	(43.7)	1/22	(4.5)	0.0054
Deaths NCPAP-MV	21/35	(60.0)	43/88	(43.9)	ns
Deaths NCPAP	5/64	(7.8)	7/233	(3.0)	ns
GA in VA deaths, N, mean ± [SD]	91	32.5 ± [4.2]	87	30.7 ± [4.1]	0.0043
BW in VA deaths, N, mean ± [SD]	92	1633 ± [774.7]	87	1383 ± [656.6]	0.0213

In the bNCPAP group deaths decreased from 7.8% in 2006 to 3.0% in 2008, but the small number of cases does not permit statistical analyses.

In VA newborns who died in 2008, however, significantly lower mean GA (30.7 *vs* 32.5 weeks) and BW (1,383 g *vs* 1,633 g) were observed compared to 2006 (Table 3).

Finally, during the two periods analysed, the stepwise logistical regression model applied to the mortality-related risk factors revealed two important inverse relationships. The first was between the probability of death and the 5-minute Apgar score (OR = 0.68, p-value <0.0001) and the second was between the probability of death and GA (OR = 0.34, p-value <0.0001). Furthermore, the model revealed that ventilation with bNCPAP decreased the risk of death compared to intubation and mechanical ventilation (ETT) (OR = 0.04, p value <0.0001), as reported in Table 4.

**Table 4:** **Logistic regression model for death risk and variables considered**

	**OR**	**95% CI**	**p**
bNCPAP *vs* ETT	0.04	(0.02 – 0.08)	<0.0001
GA z-score	0.34	(0.26 – 0.44)	<0.0001
Apgar score at 5 minutes	0.68	(0.59 – 0.79)	<0.0001

GA z-score z score of gestational age.

### NICU duration of stay and ventilation mode distribution

An increase in the mean duration of NICU stay was observed in VA patients: 14.6 days in 2006 and 17.5 days in 2008 (p = 0.0481) (Table 5).

**Table 5:** **Distribution of NICU stay and duration of ventilation**

	**2006**		**2008**		**p**
	**N**	**Mean ± [SD]**	**N**	**Mean ± [SD]**	
**Days in NICU**					
VA patients	218	14.6 ± [16.2]	376	17.5 ± [19.9]	0.0481
EET group	159	14.4 ± [16.9]	148	18.9 ± [22.3]	ns
bNCPAP group	59	15.1 ± [14.6]	228	16.9 ± [18.2]	ns
**Hours of MV**					
EET group	158	101.6 ± [113.6]	149	115.9 ± [113.9]	ns
**Hours of bNCPAP**					
All bNCPAP groups	110	49.8 ± [46.7]	336	50.5 ± [69.5]	ns

In all VA groups, the mean number of hours of the two types of assistance given (MV and bNCPAP) were calculated separately, including those of children receiving bNCPAP-MV and MV-bNCPAP. As shown in Table 5 all values were comparable during the two periods.

The overall number of MV and bNCPAP hours was calculated in the two study periods, showing a 209.6% increase in the overall bNCPAP hours in 2008 compared to 2006, due to the increase in bNCPAP newborns (Table 5).

## Discussion

In this study the interventions proposed resulted in decreased rates of intubation and mechanically ventilated newborns (72 *vs* 39%; p = 0.0001) and in a proportionally larger number of patients treated exclusively with bNCPAP (27% *vs* 61%; p = <0.0001). Mortality rate was significantly reduced (40.4 *vs* 22.7%; p = 0.0001). The average length of NICU stay in VA patients increased in 2008 to 2.9 days. There was a large increase in overall number of hours of bNCPAP (209%) in 2008 compared to 2006.

### Characteristics of the VA newborns

The BCH is the national referral centre for pathologic pregnancies. During the two periods, the characteristics of all VA patients were similar. The higher 2008 CS rate may be explained by a generally more prudent attitude towards term and preterm delivery during recent years [34,35] rather than by greater severity of cases in 2008. The relative increase in CS percentage is also likely due to hospital characteristics. Conversely, the decreased rate of newborns requiring resuscitation (54% in 2006 and 39% in 2008) and intubation at birth (25% in 2006 and 15% in 2008) may be related to the greater number of CS in 2008. It was not possible to apply a risk index [32], but no differences emerged between 2006 and 2008 in Apgar indeces in resuscitated newborns, supporting comparable clinical conditions in the two groups. Consistent with this hypothesis, the multivariate analysis showed no influence of delivery mode and newborn resuscitation on mortality. The association between death risk and Apgar score at 5 minutes, however, is well documented in the literature [36].

Steroid prophylaxis against RDS was the only available prenatal care index and was comparable in the two periods. The lack of data on other prenatal care and maternal socio-economic characteristics (instruction, wealth, parity, age, income, etc.) may be a limitation of this study. A change in prenatal care and maternal socio-economic conditions, however, is unlikely given the short study period. No differences were found between 2006 and 2008 in terms of mean GA and BW, GA, MV, and 5-minute Apgar score emerged as the most relevant factors in determining the outcome of all VA newborns.

### Ventilation mode and mortality

In this study it was not possible to evaluate MV-related lung damage, such as BPD [12,18,19], but only intubation and MV impact on mortality rate.

Multivariate analysis for the most influential death-related risk factors shows MV as one of the only three relevant variables.

In the ETT group of patients, mortality rate remained unchanged (53%), but the percentage of ETT patients decreased from 72% in 2006 to 39% during 2008, leading to a consistent reduction in the overall mortality (Figure 3). In the MV patient group, the death rate increased from 59% to 90%, with no impact on the overall mortality, due to the concurrent decrease in number of newborns in this group (from 50% to 10%).

Such high mortality rates in ventilated newborns can be explained both by the fact that the more compromised newborns received MV more frequently and by the lack of surfactant therapy.

In the groups of MV-bNCPAP patients, bNCPAP was applied after MV to stabilise patients and to avoid re-intubation [4], and the rate remained very small (6% of all VA newborns) during the periods examined, with no influence on mortality.

Patients failing to respond to the bNCPAP in either period were switched to MV (bNCPAP-MV group). Since the use of bNCPAP was systematically encouraged during 2008, a consistent increase in failure incidence (15% *vs* 23% p = 0.02) in this group was conceivable. However, the bNCPAP failure rate in the bNCPAP-MV group was limited and comparable with literature data [37,38]. As shown in Table 4 the mortality rate in this group didn’t change in the two periods. This is a clinically relevant point, since an inappropriate delay in MV is known to compromise its efficacy and the patient’s clinical outcome.

All these considerations seem to support an appropriate application of both ventilation methodologies during 2008.

The high success of the non-invasive respiratory support was also associated with its early application [12,19], as 54% of the patients started at birth and 86% within the first two hours of life. Moreover, the mean GA of newborns who died during 2008 was two weeks less than that observed in 2006, further supporting the improvement in assistance quality level.

### Duration of NICU admission and distribution of ventilation type

Mean duration of NICU stay increased by 2.9 days during 2008 in newborns from the VA group (Table 3).

Non-respiratory impairment, infection complications, nutritional factors, and preterm birth-related complications generate complexity in assistance and can increase duration of NICU stay. Despite the increase in number of VA newborns in 2008, the ETT group decreased from 72% to 39% in 2008, representing nearly an equivalent number of newborns. For this reason, the total hours of MV for the ETT group increased by only 7.6% (Table 3). Recent data have shown that the rate of serious adverse events related to MV (e.g. accidental disconnection from the respiratory circuit or unintentional removal or obstruction of the tracheal tube) is proportional to the nursing workload [39]. Assistance shifted to the bNCPAP patients, accounting for a 209% increase in the overall amount of bNCPAP hours of assistance compared to 2006. The improved survival that was observed was significant, but also let to an increase in the workload in caring for patients with bNCPAP, given the greater need for nursing staff time and expertise. A change in clinical practices therefore occurred, with an improvement in quality of care. The reduced need for MV leads to a reduction in use of resources, and this is especially important in settings with resource limitations.

### NCPAP in LMICs

Few studies evaluating NCPAP use in LMICs exist. Urs and colleagues, in a prospective observational study, obtained an 80% success rate using bNCPAP in 50 Indian preterms (28–37 weeks GA), supporting the primary use of the bNCPAP in poorer countries [40]. In a retrospective descriptive analysis from Zimbabwe, data from 234 newborns (1,730 g mean BW) with a death rate incidence of 46 · 4% were analysed to compare MV with NCPAP respiratory support. MV resulted as the only variable associated with a significant increase in death risk when compared to NCPAP [24].

In extreme conditions, the lack of resources leads to restrictive criteria for access to NICUs. In South Africa, Piepier and colleagues found that, in 21 VLBW newborns who were not admitted to the NICU, the introduction of NCPAP support led to a significant reduction in mortality rate, without severe complications. The authors also found that, given the simplicity of the method, it could be provided by trained nurses even in VLBW infants [23]. The safety and efficacy of bNCPAP, applied by trained nurses for reducing MV, was confirmed by Koyamaibole [27]. A recent Indian RCT comparing bNCPAP with ventilator NCPAP found greater success, and comparable safety, in managing preterm neonates with early onset respiratory distress with the first method [8]. Despite the fact that surfactant was listed as an essential drug by the World Health Organisation, its use in resource limited settings is a matter of debate [41]. Its cost is unaffordable for the health economy of most developing countries, including Nicaragua, and bNCPAP is an affordable and determining strategy for reducing MV and the need for surfactant therapy. The extremely low availability of surfactant is unacceptable and makes the present study somewhat comparable to the first CPAP studies, in which surfactant was not available.

Despite the fact that the published studies had limited statistical power due to their small population sizes, the accumulated evidence [3,8,9,23-27,40-42] is enough to consider NCPAP, particularly bNCPAP, as the first-line standard of care for efficacy, cost effectiveness, and ease of use in newborns with respiratory distress in LMICs [43].

## Conclusions

This is the first study to be performed in a large NICU from a developing country. It demonstrates an association between the systematic use of a low-cost bNCPAP device and a reduction in mortality and strongly supports the early use of bNCPAP as the primary respiratory assistance strategy when mechanical ventilation is needed.

The proportion of bNCPAP newborns reached 61% and mortality decreased to 22% in 2008, with no significant differences in the populations from the two time periods. Economic data were missing, but, as in other LMICs with restricted health resources and high neonatal mortality rates, Nicaragua has limited NICU facilities. The implementation of a low-cost bNCPAP strategy represents an adequate system for avoiding invasive procedures, containing the need for sophisticated technologies, and reducing economic pressure.

### Ethics approval

Institutional Review Board of the Bertha Calderon Hospital, Managua, Nicaragua and of the IRCCS – Istituto di Ricerche Farmacologiche Mario Negri, Milan, Italy.
